# The alternating access mechanism of transport as observed in the sodium-hydantoin transporter Mhp1

**DOI:** 10.1107/S0909049510032449

**Published:** 2010-11-05

**Authors:** Simone Weyand, Tatsuro Shimamura, Oliver Beckstein, Mark S. P. Sansom, So Iwata, Peter J. F. Henderson, Alexander D. Cameron

**Affiliations:** a Division of Molecular Biosciences, Membrane Protein Crystallography Group, Imperial College, London SW7 2AZ, UK; b Japan Science and Technology Agency, Exploratory Research for Advanced Technology, Human Receptor Crystallography Project, Yoshida-Konoe-cho, Sakyo-ku, Kyoto 606-8501, Japan; c Membrane Protein Laboratory, Diamond Light Source, Harwell Science and Innovation Campus, Chilton, Didcot, Oxfordshire OX11 0DE, UK; d Department of Cell Biology, Graduate School of Medicine, Kyoto University, Yoshida-Konoe, Sakyo-Ku, Kyoto 606-8501, Japan; e Department of Biochemistry, University of Oxford, Oxford OX1 3QU, UK; f Systems and Structural Biology Center, RIKEN, 1-7-22 Suehiro-cho, Tsurumi-ku, Yokohama 230-0045, Japan; g Astbury Centre for Structural Molecular Biology, Institute for Membrane and Systems Biology, University of Leeds, Leeds LS2 9JT, UK

**Keywords:** membrane transport, transport protein, alternating access, hydantoins

## Abstract

Secondary active transporters move molecules across cell membranes by coupling this process to the energetically favourable downhill movement of ions or protons along an electrochemical gradient. They function by the alternating access model of transport in which, through conformational changes, the substrate binding site alternately faces either side of the membrane. Owing to the difficulties in obtaining the crystal structure of a single transporter in different conformational states, relatively little structural information is known to explain how this process occurs. Here, the structure of the sodium-benzylhydantoin transporter, Mhp1, from *Microbacterium liquefaciens*, has been determined in three conformational states; from this a mechanism is proposed for switching from the outward-facing open conformation through an occluded structure to the inward-facing open state.

## Abstract 2

Crystal structures of a membrane protein transporter in three different conformational states provide insights into the transport mechanism.

## 1. Introduction

Membrane protein transporters facilitate the movement of small molecules across cell membranes. Many of these integral membrane proteins can be broadly categorized into two classes, primary and secondary transporters. Primary transporters are energized by the hydrolysis of ATP, redox reactions or light. Secondary transporters, on the other hand, harness the energy stored in electrochemical gradients across the membrane to the transport process (Crane *et al.*, 1961 ▶; Mitchell, 1957 ▶). They can be further subdivided into three categories. Uniporters translocate a single substrate along a concentration gradient; symporters co-transport a substrate in the same direction as ions or protons; antiporters couple the movement of one substrate in one direction with another in the opposite direction.

Many transporters are thought to work by an alternating access mechanism. The principle of this mechanism, which was first proposed in 1966 (Jardetzky, 1966 ▶), is that a substrate binding to a cavity on one side of the membrane should trigger a conformational change of the protein to allow the substrate to dissociate on the other side. This process should go through at least one intermediary state in which the substrate binding site is occluded from both sides and there should never be direct access from one side of the protein to the other as is observed in channels. This overall process is well characterized biochemically, but for secondary transporters there is a paucity of detailed structural information available to explain the mechanism. This is due to the difficulty of elucidating the structure of these relatively unstable membrane proteins in multiple states. We have been fortunate in obtaining the structure of a secondary transporter in three different conformational states and consequently are able to address this question more thoroughly (Weyand *et al.*, 2008 ▶; Shimamura *et al.*, 2010 ▶).

Mhp1 from *Microbacterium liquefaciens* is a member of the nucleobase-cation symport-1 family of transporters (Suzuki & Henderson, 2006 ▶). These transporters allow the uptake of nucleobases that can be used as energy sources or for the supply of biosynthetic precursors in bacteria and fungi (Weyland *et al.*, 2010 ▶). They are symporters that co-transport substrate from the extracellular milieu with sodium ions or protons. Mhp1 itself was discovered because it belongs to a gene cluster involving a hydantoinase and carbamoylase that convert 5-substituted l-hydantoins into l-amino acids (Suzuki & Henderson, 2006 ▶). It was found to catalyse the transport of 5-indolylmethyl- or 5-benzylhydantoin into the bacteria, where they can be converted to tryptophan or phenyl alanine by the other enzymes of the gene cluster.

## 2. Structure of Mhp1

**Figure 1:**
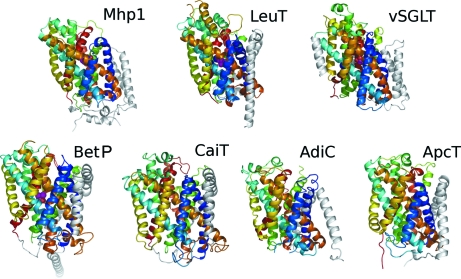
Comparison of Mhp1 with the structures of other transporters with the same fold. The core ten helices of each protein have been coloured according to residue number starting with red at the N-terminus going through the colours of the rainbow and finishing with blue at the C-terminus. The TMs that are extra to this core have been coloured grey. The first three panels of this figure are from Weyand *et al.* (2008 ▶).

**Figure 2:**
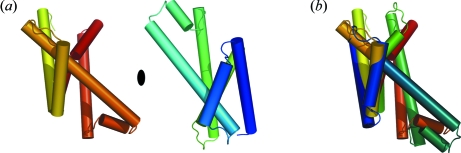
The inverted repeat of Mhp1. (*a*) The N- and C-terminal repeat units are shown in the same orientation and same colouring as seen in Fig. 1 ▶ but they have been separated for emphasis. The position of the twofold pseudo axis is denoted by an oval. (*b*) The C-terminal repeat has been rotated around the pseudo-twofold axis seen in (*a*).

The crystal structure of Mhp1 was solved initially at a resolution of 2.85 Å (Weyand *et al.*, 2008 ▶). It is composed of 12 transmembrane helices (TMs) (Fig. 1 ▶). The first ten TMs are arranged in a manner that was first seen for the bacterial homologue of the serotonin transporter LeuT (Yamashita *et al.*, 2005 ▶), but has now been observed in a number of other structures published in the last three years. These structures span numerous secondary transporters from diverse families that, owing to their low sequence similarity, were not expected to have a similar fold. In addition to LeuT from the neurotransmitter-sodium-symport (NSS) family and Mhp1 from the nucleobase cation symport-1 (NCS1) family, these include the sodium-galactose symporter vSGLT (Faham *et al.*, 2008 ▶) from the sodium-solute symporter (SSS) family, the sodium-betaine symporter BetP (Ressl *et al.*, 2009 ▶) from the betaine/choline/carnitine transporter (BCCT) family, CaiT (Tang *et al.*, 2010 ▶), an antiporter from the same family, and two members of the amino acid/polyamine/organocation (APC) family AdiC (Fang *et al.*, 2009 ▶; Gao *et al.*, 2009 ▶, 2010 ▶) and ApcT (Shaffer *et al.*, 2009 ▶). This fold has been described as a five-helix inverted repeat in which the N-terminal five TMs are topologically equivalent to the second five and are related to one another by a pseudo twofold axis that runs through the centre of the membrane (Abramson & Wright, 2009 ▶) (Fig. 2 ▶). The two halves of the protein are completely intertwined with both contributing to the binding site, which is situated buried in the membrane approximately at the centres of the respective proteins.

**Figure 3:**
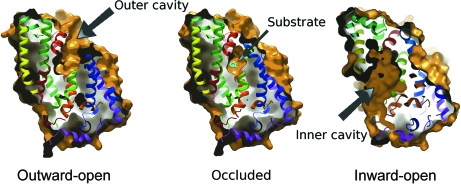
Surface representations of the three forms of Mhp1, showing the cavities in each. The ribbon diagrams have been coloured as in Fig. 1 ▶. The figure has been taken from the supplementary material of Shimamura *et al.* (2010 ▶).

In the initial structure of Mhp1 a large cavity was present extending from the extracellular face of the protein into the centre, *i.e.* the structure was outward-open in the definition of the alternating access model (Fig. 3 ▶) and did not contain bound substrate. It did, however, contain electron density at the same position as a sodium ion in LeuT, which was solved at 1.65 Å resolution (Yamashita *et al.*, 2005 ▶). Consequently this was also assigned to be sodium in Mhp1, taking into account the distances between the sodium and the surrounding residues. To obtain the occluded state we co-crystallized Mhp1 in the presence of benzylhydantoin. The subsequent structure, though at low resolution (4 Å), showed benzylhydantoin to bind at the bottom of the cavity that was observed in the outward-open structure. Concomitant with the binding a conformational change had also occurred to lock the substrate in its binding site, effectively sealing its exit to the exterior (Fig. 3 ▶). The passage to the interior was still blocked by amino acids spanning approximately 20 Å suggesting that a much larger conformational change was needed to switch the substrate binding site to be accessible to the inside. The inward-open form was obtained from crystals grown from selected batches of protein that had been expressed in *E. coli* in defined media in the presence of selenomethionine (Shimamura *et al.*, 2010 ▶). In this structure the extracellular side of the protein was completely closed and instead a cavity was present on the intracellular side. At the sodium binding site electron density was observed suggesting that an unidentified molecule had bound during the expression of the protein and locked the protein in an inward-open conformation.

## 3. Conformational changes

**Figure 4:**
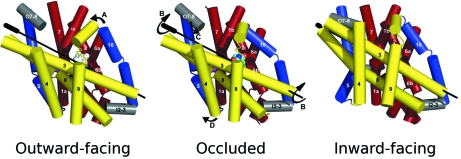
The mechanism of Mhp1 taken from Shimamura *et al.* (2010 ▶). The movements are delineated by arrows. A: Helix 10 bends over the substrate. B: The hash motif rotates by 30° around the rotation axis shown as a black line. C: The small extracellular helix moves to seal completely the extracellular side of the protein. D: Helix 5 bends to open the cavity on the intracellular side in a reciprocal manner to helix 10.

The conformational changes that take place as the protein goes from outward-open through the occluded to the inward-open state can be described best by considering the core ten TMs in three parts (Fig. 4 ▶) (Shimamura *et al.*, 2010 ▶). TMs 1 and 2 from the N-terminal repeat and TMs 6 and 7, their counterparts from the second, form a four helix bundle (the bundle, coloured red in Fig. 4 ▶). TMs 1 and 6 are both extended helices with the binding site situated where the helices break. TMs 3 and 4 and their equivalents in the C-terminal half, TMs 8 and 9, form a motif that has the appearance of the hash sign (#) (hash motif, coloured yellow) with TMs 3 and 8 running antiparallel to one another across the face of the bundle. TMs 5 and 10 are both seen to bend as the protein changes conformation (flexible helices, coloured blue). These helices, along with TMs 4 and 9, form a V and an inverted-V structure, respectively, around TMs 3 and 8 on either side of the protein (Figs. 2 ▶ and 4 ▶).

In going from the outward-open structure to the occluded structure the N-terminal half of TM 10 bends, closing over the substrate in its binding site. At this resolution there are no other significant movements that can be assigned unambiguously. However, there is a small but definite shift of the hash motif and C-terminus towards the bundle. To switch the substrate binding site from facing outward to facing inward a surprisingly simple rigid body rotation of 30° of the hash motif relative to the bundle occurs around an axis roughly in line with TM 3. This simultaneously blocks further the substrate binding site from the outside and opens it to the inside. This rotation is accompanied by two other main changes to the protein. Firstly, TM 5, the N-terminal equivalent of TM 10, flexes in a similar manner to TM 10 to open the cavity further to the exterior. Secondly, a small extracellular helix moves to seal the extracellular face of the protein completely.

## 4. Gating system

The mechanism of converting between the different states of the protein has also been described in terms of two sets of gates: thick and thin (Krishnamurthy *et al.*, 2009 ▶; Abramson & Wright, 2009 ▶). The thin gates, consisting of only a few protein residues, control the flow of substrate into and out of the cavity at either side of the protein. In Mhp1, TMs 5 and 10 perform the role of intracellular and extracellular thin gate, respectively, although at present it is unclear whether there could be an inward-facing occluded state. The thick gate affects the switching between the inward-and outward-facing states. In Mhp1 this works more like a kissing gate, where the gate is either in one conformation or the other, although unlike a normal kissing gate there is never a state in which there is a channel from one side of the protein to the other. The mechanism uses the internal symmetry of the protein with TMs 5 and 10 performing a similar function on the inside and outside of the protein, respectively. However, since the rotation axis is not in the centre of the protein and the C-terminal transmembrane helices constrain the movements in one part of the protein with respect to the other, the system is not completely symmetrical.

## 5. Sodium and substrate binding

**Figure 5:**
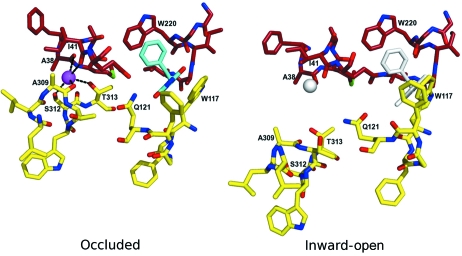
The sodium and substrate binding sites in the occluded and inward-open structures taken from Shimamura *et al.* (2010 ▶). The carbon atoms of the amino acids have been coloured as in Fig. 4 ▶. The sodium ion is represented as a magenta sphere and the benzylhydantoin with cyan carbon atoms in the occluded structure. In the inward-open structure where these entities are not present they are represented in white.

The sodium and substrate binding sites are located at the interface of the bundle and the hash motif. In the outward-facing structures both are intact with the sodium ion interacting with residues on TM 1 and TM8 and the benzyl­hydantoin fitting snugly between the indole rings of Trp 117 on TM 3 and Trp 220 in TM 6 (Fig. 5 ▶). In the inward-open structure both binding sites are disrupted, particularly the sodium binding site where the residues interacting with the ion move approximately 4.5 Å further apart (Fig. 5 ▶). The position of the sodium binding site suggests a plausible role for the ions in the mechanism. The concentration of sodium is likely to be much higher outside of the bacteria relative to the inside (Harold & Maloney, 1996 ▶). Sodium binding should stabilize the outward-facing conformation of the protein, which in the absence of sodium should only occur transiently. In this conformation the substrate-binding site is primed to accept the substrate, which is likely to be present at much lower levels. In fact, in fluorescence quenching experiments the presence of sodium increases the apparent affinity of Mhp1 for benzylhydantoin by about tenfold (Weyand *et al.*, 2008 ▶). Substrate binding should trigger a conformational change to switch the protein to the inward-facing state, so destabilizing the sodium and substrate binding sites and allowing entry of these entities into the cell. The exact steps along the pathway need to be investigated to test these proposals. Since the transport of benzylhydantoin is directly coupled with sodium it would seem that substrate binding in the absence of sodium should not trigger the switch to the inward-facing conformation. Again fluorescence quenching experiments show that the apparent affinity for sodium is increased when benzyl­hydantoin is present, suggesting that the binding of benzylhydantoin also pushes the equilibrium in favour of the outward-facing conformation. This mechanism is supported by molecular dynamics simulations (Shimamura *et al.*, 2010 ▶).

## 6. Relevance to other LeuT superfamily members

The derivation of the mechanism above was enabled by our knowledge of the three structures of Mhp1. Since Mhp1 is part of the LeuT superfamily the question arises as to whether this mechanism is also relevant for the other members. This superfamily contains proteins with very different substrates that are regulated by sodium or by protons and both symporters and antiporters. For the sodium-coupled sym­porters it seems likely that the switching between the outward- and inward-facing structures is based on the same principle. A similar mechanism of the switching between outward- and inward-facing conformations caused by a rigid-body movement of the four-helix bundle relative to the rest of the protein was, in fact, first proposed for LeuT based on the asymmetry of the crystal structure and investigated more thoroughly using a mutational analysis of the serotonin transporter (Forrest *et al.*, 2008 ▶). The inward-facing structure of vSGLT and the outward-facing structure of LeuT are also consistent with the mechanism (Faham *et al.*, 2008 ▶; Yamashita *et al.*, 2005 ▶). Can the mechanism be extended to the members of the APC or BCCT families? Owing to low sequence homology it is difficult to give an unambiguous answer, but by comparing the outward-facing AdiC with the inward-facing ApcT from the APC family or the occluded BetP with the inward-facing CaiT from the BCCT family the same trend can be observed with the hash motif moving relative to the bundle. The details of the conformational changes will, of course, vary from one protein to another as these proteins have very different substrates. In LeuT, for instance, the occlusion of the substrate in the binding site appears to be affected by the side chains of a few residues (Yamashita *et al.*, 2005 ▶). Conversely, in AdiC, TM 5 bends less and instead TMs 2 and 6 of the bundle adopt a new conformation (Gao *et al.*, 2010 ▶). Thus the mechanism for Mhp1 provides a framework for investigating the conformational changes of the other superfamily members, but the details of these proteins need to be investigated more thoroughly. Indeed, there is much still to do in elucidating the mechanism of Mhp1, where higher-resolution structures and mutational analysis combined with kinetic measurements are needed if we are to really understand it.
